# Chromosome Doubling of Microspore-Derived Plants from Cabbage (*Brassica oleracea* var. *capitata* L.) and Broccoli (*Brassica oleracea* var. *italica* L.)

**DOI:** 10.3389/fpls.2015.01118

**Published:** 2015-12-22

**Authors:** Suxia Yuan, Yanbin Su, Yumei Liu, Zhansheng Li, Zhiyuan Fang, Limei Yang, Mu Zhuang, Yangyong Zhang, Honghao Lv, Peitian Sun

**Affiliations:** Institute of Vegetables and Flowers, Chinese Academy of Agricultural SciencesBeijing, China

**Keywords:** cabbage (*Brassica oleracea* var. *capitata* L.), broccoli (*Brassica oleracea*. var. *italica* L.), microspore-derived plants, chromosome doubling, ploidy determination

## Abstract

Chromosome doubling of microspore-derived plants is an important factor in the practical application of microspore culture technology because breeding programs require a large number of genetically stable, homozygous doubled haploid plants with a high level of fertility. In the present paper, 29 populations of microspore-derived plantlets from cabbage (*Brassica oleracea* var. *capitata*) and broccoli (*Brassica oleracea* var. *italica*) were used to study the ploidy level and spontaneous chromosome doubling of these populations, the artificial chromosome doubling induced by colchicine, and the influence of tissue culture duration on the chromosomal ploidy of the microspore-derived regenerants. Spontaneous chromosome doubling occurred randomly and was genotype dependent. In the plant populations derived from microspores, there were haploids, diploids, and even a low frequency of polyploids and mixed-ploidy plantlets. The total spontaneous doubling in the 14 cabbage populations ranged from 0 to 76.9%, compared with 52.2 to 100% in the 15 broccoli populations. To improve the rate of chromosome doubling, an efficient and reliable artificial chromosome doubling protocol (i.e., the immersion of haploid plantlet roots in a colchicine solution) was developed for cabbage and broccoli microspore-derived haploids. The optimal chromosome doubling of the haploids was obtained with a solution of 0.2% colchicine for 9–12 h or 0.4% colchicine for 3–9 h for cabbage and 0.05% colchicine for 6–12 h for broccoli. This protocol produced chromosome doubling in over 50% of the haploid genotypes for most of the populations derived from cabbage and broccoli. Notably, after 1 or more years in tissue culture, the chromosomes of the haploids were doubled, and most of the haploids turned into doubled haploid or mixed-ploidy plants. This is the first report indicating that tissue culture duration can change the chromosomal ploidy of microspore-derived regenerants.

## Introduction

Microspore culture is an effective alternative technique for the production of doubled haploid (DH) parental lines to generate F_1_ hybrids (Abercrombie et al., 2005). The development of DH lines accelerates the plant breeding process by saving time and labor (Ferrie and Caswell, 2011). In addition, DH lines can also be used for marker identification, gene mapping and various genetic manipulations (Forster et al., 2007; Ferrie and Möllers, 2011; Ferrie and Caswell, 2011). Consequently, this technique has been successfully used in cabbage and broccoli, and large-scale DH lines have been developed (Cao et al., 1990; Takahata and Keller, 1991; Duijs et al., 1992; Hansen, 1994; Pink, 1999; da Silva Dias, 2003; Yuan et al., 2009, 2011, 2012). Furthermore, some DH parental lines have even been introduced into breeding schemes (Hale et al., 2007; Lv et al., 2014). The procedure for DH production includes two major steps: haploid induction and chromosome doubling. Consequently, the chromosome doubling of haploids derived from microspores is an important step in the practical application of microspore culture technology. Microspore-derived haploids can spontaneously double their chromosomes during the very early stages of embryogenesis or can be induced to become DHs in the later stages of development (Palmer et al., 1996).

The ideal goal is to double the chromosome number of the original microspore and to then regenerate a plant from the resulting DH microspore. Theoretically, this would result in a stable, homozygous, completely fertile DH. Presently, the mechanism underlying this spontaneous chromosome doubling is unclear in many instances, with wide differences in the responses among and within species (Kasha, 2005). da Silva Dias (2003) and his group found 43–88% spontaneous diploids in broccoli and 7–91% spontaneous diploids in other coles. Because spontaneous doubling occurs randomly and is extremely genotype-dependent, it is important to ascertain the level of spontaneous diploids in the genotype used. da Silva Dias (2003) suggested that for genotypes with over 60% spontaneous doubling, it was not necessary to induce doubling. Obviously, for those genotypes with low spontaneous doubling rates, a successful chromosome-doubling process is essential for the production of homozygous plants after haploid plants are derived from the microspores of plants such as cabbage and broccoli. Various doubling agents have been studied, including caffeine (Thomas et al., 1997), nitrous oxide (Hansen et al., 1988), and the antimicrotubule herbicides trifluralin and amiprophos-methyl (APM; Hansen and Andersen, 1998b). However, the most commonly used chemical agent for chromosome doubling is colchicine (Niu et al., 2014), which disrupts mitosis by inhibiting the formation of spindle fibers and disturbing normal polar chromosomal migration, resulting in chromosome doubling (Jensen, 1974). da Silva Dias (2003) found that when the roots of plants derived from microspores were immersed in a 0.25% colchicine solution, a 53–71% doubling rate was achieved.

Previous studies examining the techniques of microspore culture in cole crops were focused strongly on improving embryogenesis and plant regeneration but ignored research on chromosome doubling. To date, successful microspore culture techniques have been established for cabbage and broccoli (Hansen, 2003; da Silva Dias, 2003; Yuan et al., 2009, 2011, 2012). Flower buds containing a large number of late uninucleate stage microspores and about 10–30% binucleate microspores were selected for microspore cultures in cabbage and broccoli (da Silva Dias, 2003). Heat shock pretreatment was shown to induce microspore embryogenesis in *Brassica oleracea* (Takahata and Keller, 1991; Duijs et al., 1992; da Silva Dias, 2001). In our previous research, the combination of cold pretreatment (4°C) for 1 or 2 days and heat shock (32.5°C) for 1 day significantly enhanced microspore embryogenesis in broccoli (Yuan et al., 2011), and 32.5°C for 1 or 2 days was optimal in cabbage (Yuan et al., 2012). NLN-13 medium and ½ NLN-13 medium were efficient on microspore embryogenesis in cabbage (Duijs et al., 1992) and broccoli (da Silva Dias, 2001), respectively. Furthermore, da Silva Dias (1999) reported that the addition of activated charcoal increased significantly embryo yields in nine genotypes of *B. oleracea.* Our previous study indicated that the combination of 10 mg l^−1^ gum arabic and 3 mM MES in NLN-13 at pH 6.4 significantly enhanced microspore embryogenesis in cabbage (Yuan et al., 2012). Based on the above-mentioned research, a large number of microspore-derived plant in cabbage and broccoli were obtained (Duijs et al., 1992; Yuan et al., 2009). For the useful application of microspore culture techniques in cabbage and broccoli breeding programs, the chromosome doubling of plants derived from microspores must be investigated and improved.

In the present paper, we used 29 populations of microspore-derived plantlets from cabbage (*Brassica oleracea* var. *capitata*) and broccoli (*Brassica oleracea* var. *italica*) to study the ploidy levels and spontaneous chromosome doubling in these populations. Additionally, we examined artificial chromosome doubling induced by colchicine and the influence of tissue culture duration on the chromosomal ploidy of the microspore-derived regenerants. Our objectives were to ascertain the spontaneous doubling of the populations, to assess the impact of tissue culture duration on chromosome doubling in the populations and to develop an efficient and reliable artificial chromosome doubling protocol for microspore-derived haploids of cabbage and broccoli.

## Materials and methods

### Plant materials

From 2005 to 2013, the microspore culture of cabbage (*Brassica oleracea* var. *capitata*) and broccoli (*Brassica oleracea* var. *italica*) was undertaken from March to May at the Institute of Vegetables and Flowers, Chinese Academy of Agricultural Sciences, Beijing, China. The microspore isolation and culture procedures were performed as previously described by Yuan et al. (2011, 2012). Briefly, NLN-13 (Yuan et al., 2012) and ½ NLN-13 (da Silva Dias, 2001) were used as liquid microspore culture media for cabbage and broccoli, respectively. The microspores were incubated in the dark at 32.5 ± 1°C for 1 day and then maintained at 25 ± 1°C in the dark. Cotyledonary embryos were obtained from 24-day-cultured microspores. The embryo culture and plant regeneration were carried out according to the procedures described by Yuan et al. (2011). During subsequent tissue culture, the plantlets were subcultured using fresh solid MS-2 medium (Murashige and Skoog, 1962; 2% sucrose, 0.5% agar, 0.1 mg/l 1-naphthaleneacetic acid (NAA), 0.2 mg/l 6-benzylaminopurine (BAP), pH 5.8) every 2 months. A total of 29 populations of cabbage and broccoli microspore-derived plantlets were obtained (Table 1). One copy of the microspore-derived plants was continually grown in a tissue culture room, while another copy was transferred to a greenhouse after October and then planted in a field to allow flowering and set seed.

**Table 1:** **Microspore-derived cabbage and broccoli plantlet populations with different genotypes were obtained from 2005 to 2013**.

**Population genotype**		**Year**	**Population genotype**		**Year**
Cabbage	Zhonggan No. 11	2005~2007, 2009	Broccoli	Lüxiu	2005~2007
	8398	2005~2007		Lüxing	2005~2007
	Xiwang	2005~2007		TI-111	2005~2007
	Zhonggan No. 18	2005~2007		TI-089	2005~2007
	08F8	2009		Lüyu	2005~2007
	08F9	2009		Yuguan	2005~2007
	08F15	2009		09B411	2010
	08F16	2009		09B524	2010
	08F17	2009		10B635	2011
	08F18	2009		10B644	2011
	08F24	2009		10B665	2011
	08F25	2009		10B908	2011
	Gen 165	2012		10B1092	2011
				12B520	2013
				12B1153	2013

### Chromosome doubling

#### Spontaneous doubling

To accurately determine the chromosomal ploidy of the regenerants, the ploidy level of each population mentioned above was identified using two methods: chloroplast counting and morphological identification. Chloroplast counting was used for early ploidy identification in tissue culture, and then to divide each population into haploid group, haploid group, polyploid group and ploid-mixed group in order to facilitate morphological identification in flowering.

#### Artificial chromosome doubling

The haploid genotype 04M1-93 derived from cabbage “Zhonggan No. 11” and the haploid genotype 05B743-49 derived from broccoli “TI-111” were tested in this experiment. To obtain sufficient plants for artificial chromosome doubling research, the two haploids were propagated in MS medium (Murashige and Skoog, 1962; 3% sucrose, 0.5% agar, 0.1 mg/l NAA, 1 mg/l BAP, pH 5.8). In the mid of September, 2007, all of the haploid plants were cut free from the hypocotyl tissue and were transferred to glass growth vessels containing solid ½ MS-2 medium (Murashige and Skoog, 1962; the concentration of the major salts was reduced to 50% compared with MS medium, 2% sucrose, 0.5% agar, 0.1 mg/l NAA, 0.1 mg/l indole-3-butytric acid (IBA), and pH 5.8), in which rooting took place.

The colchicine treatment was as follows: 2 weeks after rooting, the rooted plants were removed from the medium and washed completely with warm tap water; the roots were then trimmed to a length of 1–2 cm and immersed in a working solution of colchicine (supplemented with 2% DMSO). The plants were placed under intense light at 25°C. Next, the solution was poured off, and the roots were rinsed thoroughly in tap water. The treated plants were replanted in a soil mixture (peat soil: perlite: vermiculite, 8:1:1) in a pot and maintained for approximately 2 more weeks in a room at 24°C with a 16-h photoperiod, with low light intensity and high humidity. This was followed by gradual adaptation to greenhouse conditions. Approximately 6 weeks later, the plants were transferred to a cold frame for the duration of the winter. In April of the next year, the plants started flowering, and the ploidy level of the plants was determined using morphological identification.

In this experiment, four concentration levels of colchicine solution were tested: 0.05, 0.1, 0.2, and 0.4%. The plants were treated for 3, 6, 9, and 12 h, in each colchicine solution. Fifteen plants of every haploid genotype were used in each treatment. The same number of plants of each haploid genotype grown without colchicine served as the controls.

#### Evaluation of artificial chromosome doubling

Based on the experimental results of 2.2.2, artificial chromosome doubling induced by colchicine was applied to 576 haploid genotypes derived from six cabbage and six broccoli populations to test the chromosome doubling efficiency. In this experiment, 4–8 plants of each haploid genotype, i.e., a total of 3128 haploid plants, were treated with a colchicine solution.

### Determination of the ploidy level

#### Chloroplast number

In the tissue cultures, chloroplast counting (Yuan et al., 2009) was used to determine the initial ploidy level of the populations. In cole crops, haploid plants have at most 10 chloroplasts, diploid plants have 11–15, and polyploid plants have more than 15 chloroplasts.

#### Morphological identification

In the mid of September each year, another copy of each of the above-mentioned microspore-derived plantlets obtained in the same year was cut free from the hypocotyl tissue and transferred to a glass growth vessel containing solid ½ MS-2 medium, in which rooting took place. After 2 weeks, the rooted plantlets were transferred to a soil-perlite mixture in a pot. This transfer was then followed by the gradual adaptation to greenhouse conditions. At the end of January of the following year, the plantlets were transferred to a cold frame for the winter. In April of that year, the regenerated plants began flowering, and the population ploidy level was determined using morphological identification.

**Figure 1:**
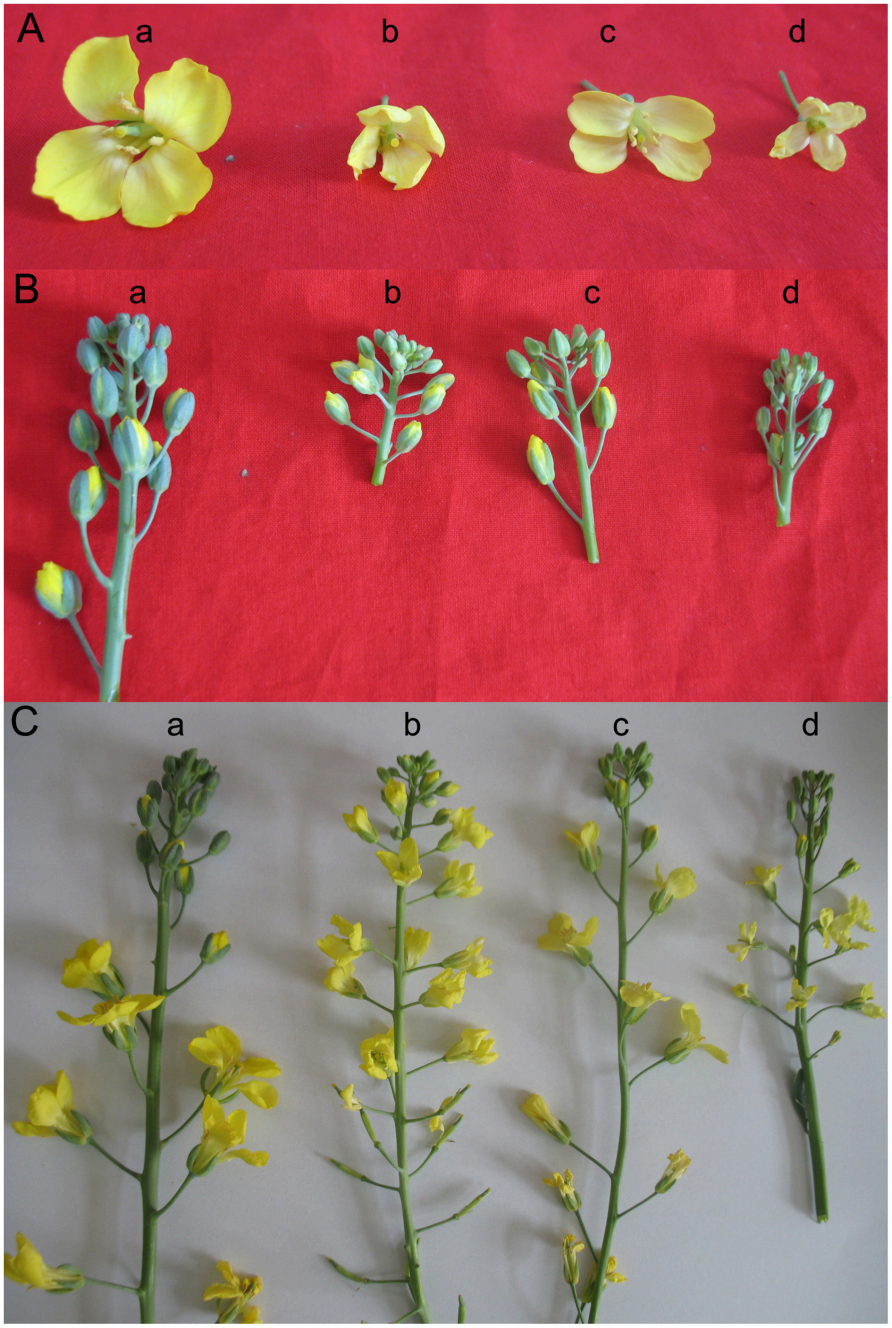
**The characteristics of the inflorescences, buds and flowers from different chromosome ploidy plants derived from cabbage “Zhonggan No. 11” microspores. (A)** Flowers from different chromosome ploidy plants; **(B)** Buds from different chromosome ploidy plants; **(C)** Inflorescences from different chromosome ploidy plants; (a) Tetraploid; (b) Triploid; (c) Diploid; (d) Haploid.

The sizes of the plants, buds and flowers were observed in the field from 2006 to 2014. The presence or absence of pollen was an additional morphological feature that was determined. Normal diploid cabbage and broccoli plants were used as controls. The characteristics of the regenerated plants with different ploidy levels were described according to the following (Figure 1):

Haploid: The growth potential of the plant is weaker, and the plant size is smaller. The flower buds are smaller, flatter and without pollen. The stamens are missing, or the stamen development is not normal.
Diploid: The plant grows normally and has pollen. The stamens and pistils are normal.
Triploid: The flower buds are smaller, flatter and without pollen. The stamens are missing, or the stamen development is not normal.
Tetraploid: The plant has pollen and stronger growth vigor. The plant size and flower buds are larger. The stamens and pistil are normal.

### Data analysis

The data were analyzed using Microsoft Excel 2003.

## Results

### Ploidy level and spontaneous chromosome doubling of the populations

**Figure 2:**
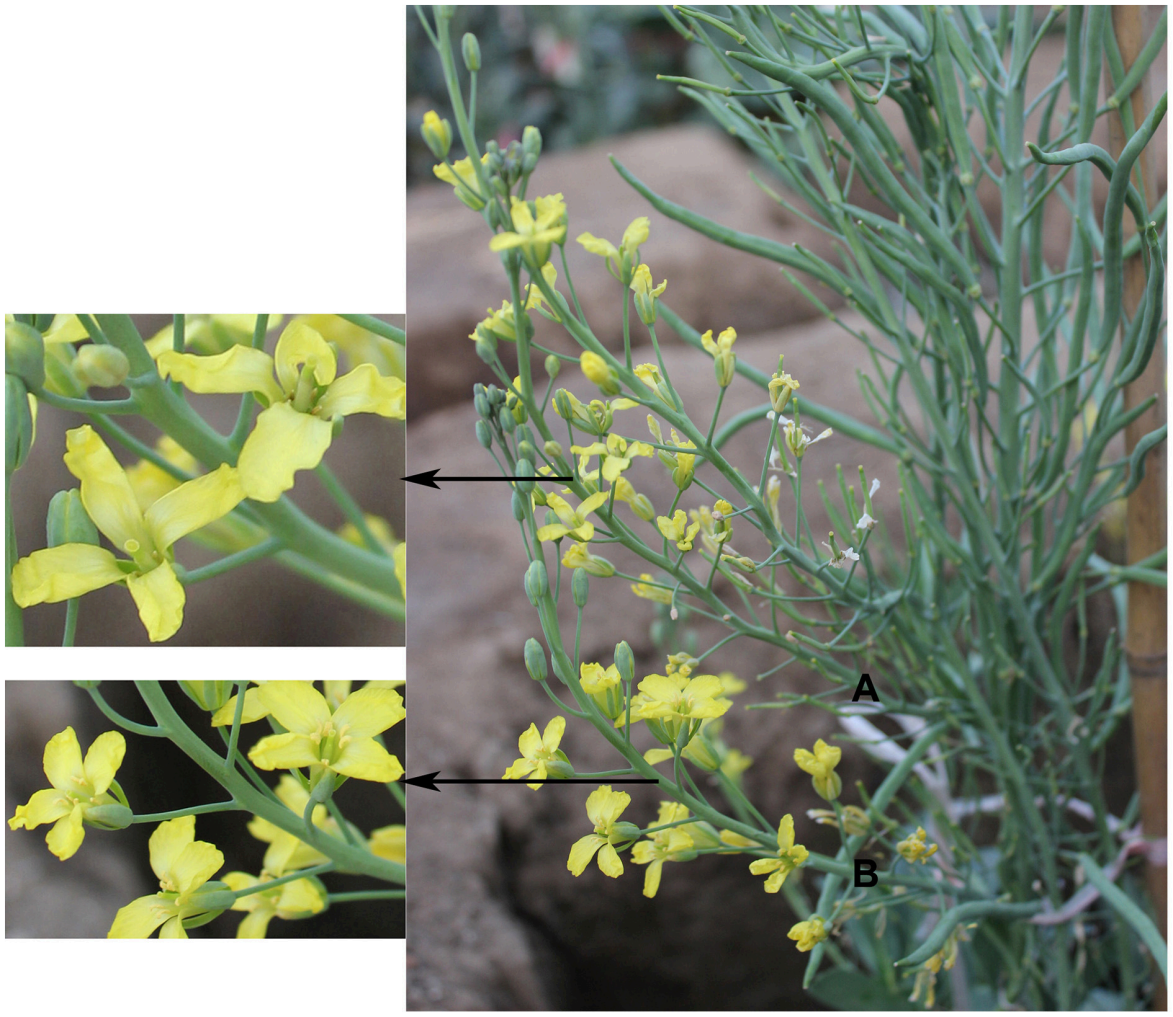
**The morphological characteristics of mixed-ploidy plants (a plant having both haploid and diploid branches simultaneously) derived from cabbage “Zhonggan No. 11” microspores. (A)** Flowers from a haploid branch; **(B)** Flowers from a diploid branch.

In total, 1717 regenerants of cabbage and 622 of broccoli were derived and investigated for their ploidy level (Table 2). Spontaneous doubling occurred randomly and was extremely genotype dependent. In the microspore-derived populations, in addition to haploids and diploids, there was a low frequency of polyploids and mixed-ploidy plantlets (a plant having both haploid and diploid branches simultaneously; Figure 2). For the 14 cabbage genotypes, the spontaneous diploid rates for the populations were in the range of 0–76.9%, and the total spontaneous doubling (including diploids, polyploids, and mixed-ploidy plantlets) ranged from 0 to 84.6%. By contrast, in the 15 broccoli populations, the spontaneous diploid rate was 50.6–100%, and the total spontaneous doubling ranged from 52.2 to 100%. In this experiment, cabbage exhibited a larger variation in the rate of spontaneous doubling in the microspore-derived regenerated populations than did broccoli.

**Table 2:** **Chromosomal ploidy levels of cabbage and broccoli populations derived from microspores**.

**Materials**		**Number of populations**	**Haploid**		**Diploid**		**Polyploid**		**Mixed-ploidy**		**Rate of chromosome doubling (%)**
			**Number of plants**	**Frequency (%)**	**Number of plants**	**Frequency (%)**	**Number of plants**	**Frequency (%)**	**Number of plants**	**Frequency (%)**	
Cabbage	Zhonggan No. 11	210	35	16.7	158	75.2	2	1.0	15	7.1	83.3
	8398	381	205	53.8	125	32.8	5	1.3	46	12.1	46.2
	Xiwang	52	8	15.4	40	76.9	0	0	4	7.7	84.6
	Zhonggan No. 8	53	11	20.8	38	71.7	1	1.9	3	5.6	79.2
	Zhonggan No. 21	34	22	64.7	10	29.4	2	5.9	0	0.0	35.3
	08F8	106	52	49.1	49	46.2	2	1.9	3	2.8	50.9
	08F15	3	3	100.0	0	0.0	0	0.0	0	0.0	0.0
	08F16	2	2	100.0	0	0.0	0	0.0	0	0.0	0.0
	08F17	3	2	66.7	1	33.3	0	0.0	0	0.0	33.3
	08F9	116	95	81.9	12	10.3	4	3.5	5	4.3	18.1
	08F18	99	31	31.3	62	62.6	4	4.0	2	2.0	68.7
	08F24	380	171	45.0	192	50.5	3	0.8	14	3.7	55.0
	08F25	266	123	46.2	124	46.6	11	4.2	8	3.0	53.8
	gen 165	12	12	100.0	0	0.0	0	0.0	0	0.0	0.0
Broccoli	Lüxiu	23	11	47.8	12	52.2	0	0.0	0	0.0	52.2
	Lüxing	163	8	4.9	150	92.0	1	0.6	4	2.5	95.1
	TI-111	91	42	46.1	46	50.6	0	0.0	3	3.3	53.9
	TI-089	20	0	0.0	20	100.0	0	0.0	0	0.0	100.0
	Lüyu	152	4	2.6	144	94.7	1	0.7	3	2.0	97.4
	Yuguan	3	0	0.0	3	100.0	0	0	0	0.0	100.0
	09B411	32	6	18.8	23	71.9	1	3.1	2	6.2	81.2
	09B524	4	0	0.0	4	100.0	0	0.0	0	0.0	100.0
	10B908	36	16	44.4	19	52.8	0	0.0	1	2.8	55.6
	10B1092	36	14	38.9	21	58.3	0	0.0	1	2.8	61.1
	10B635	24	5	20.8	19	79.2	0	0.0	0	0.0	79.2
	10B644	21	5	23.8	15	71.4	1	4.8	0	0.0	76.2
	10B665	2	0	0.0	2	100.0	0	0.0	0	0.0	100.0
	12B520	12	3	25.0	9	75.0	0	0.0	0	0.0	75.0
	12B1153	3	0	0.0	3	100.0	0	0.0	0	0.0	100.0

### Artificial chromosome doubling induced by colchicine

The data presented in Table 3 indicate that the plant survival rate gradually decreased with an increase in the concentration of the colchicine solution. Similarly, for the same concentration of colchicine, the plant survival rate decreased with an increase of the treatment time. Therefore, a higher concentration of colchicine or a longer duration of colchicine treatment had negative effects on the survival of the haploids; this phenomenon was more obvious in broccoli than in cabbage. In addition, colchicine not only induced the doubling of haploids but also induced a certain frequency of haploids to become mixed-ploidy plants (Figure 2).

**Table 3:** **Efficiency of chromosome doubling of different haploids induced by colchicine**.

**Materials**		**Concentration of colchicine (%)**	**Treatment duration (h)**	**Number of plants**	**Number of surviving plants**	**Chromosomal ploidy level**			**Frequency of chromosome doubling (%)**		**Survival rate (%)**
						**Haploid**	**Diploid**	**Mixed-ploidy**	**Diploid frequency**	**Total chromosome doubling**	
Cabbage	04M1-93	0 (CK)	0	15	15	15	0	0	0.0	0.0	100.0
		0.05	3	15	15	12	0	3	0.0	20.0	100.0
			6	15	15	12	0	3	0.0	20.0	100.0
			9	15	15	7	0	8	0.0	53.3	100.0
			12	15	15	7	1	7	6.7	53.3	100.0
		0.10	3	14	14	10	0	4	0.0	28.6	100.0
			6	15	15	9	0	6	0.0	40.0	100.0
			9	14	14	4	3	7	21.4	71.4	100.0
			12	15	15	8	4	3	26.7	46.7	100.0
		0.20	3	15	15	5	0	10	0.0	66.7	100.0
			6	15	13	7	3	3	23.1	46.2	86.7
			9	15	12	5	6	1	50.0	58.3	80.0
			12	15	12	3	3	6	25.0	75.0	80.0
		0.40	3	15	14	3	5	6	35.7	78.6	93.3
			6	15	13	2	3	8	23.1	84.6	86.7
			9	15	11	2	3	6	27.3	81.8	73.3
			12	15	6	3	0	3	0.0	50.0	40.0
Broccoli	05B743-49	0 (CK)	0	4	4	4	0	0	0.0	0.0	100.0
		0.05	3	15	14	8	4	2	28.6	42.9	93.3
			6	15	11	5	3	3	27.3	54.5	73.3
			9	14	8	2	3	3	37.5	75.0	57.1
			12	15	12	5	2	5	16.7	58.3	80.0
		0.10	3	15	13	7	1	5	7.7	46.2	86.7
			6	15	6	1	1	4	16.7	83.3	40.0
			9	15	6	1	1	4	16.7	83.3	40.0
			12	15	3	0	2	1	66.7	100.0	20.0
		0.20	3	15	7	2	1	3	14.3	57.1	46.7
			6	15	6	2	3	1	50.0	66.7	40.0
			9	15	2	0	0	1	0.0	50.0	13.3
			12	15	2	0	1	1	50.0	100.0	13.3
		0.40	3	15	3	1	1	1	33.3	66.7	20.0
			6	15	1	0	0	1	0.0	100.0	6.7
			9	15	0	0	0	0	0.0	0.0	0.0
			12	15	0	0	0	0	0.0	0.0	0.0

The 0.2% colchicine solution produced negative effects on the survival of the 04M1-93 haploids derived from “Zhonggan No. 11” cabbage; however, the 0.05% colchicine solution produced negative effects on the survival of the 05B743-49 haploids derived from broccoli “TI-111.”

When the cabbage haploids were treated with the 0.2% colchicine solution for 9 h, the DH frequency was the highest, up to 50%, followed by the 0.4% colchicine solution for 3 h. However, when the haploids were treated with the 0.4% colchicine solution for 6 h, the total chromosome doubling (including diploids and mixed-ploidy plantlets) was the highest, followed by the 0.4% colchicine for 9 h. Mixed-ploidy plants have diploid branches, the flowers of which exhibit normal fertility and can be pollinated; consequently, these plants have value for breeding. Considering the DH frequency, survival rate and total chromosome doubling, we concluded that a better chromosome doubling effect was obtained when the haploid plants were treated with 0.2% colchicine for 9–12 h or 0.4% colchicine for 3–9 h. The DH frequency ranged from 23.1 to 50.0%, the total chromosome doubling ranged from 58.3 to 84.6%, and the survival rate ranged from 73.3 to 93.3%.

Similarly, for the 05B743-49 haploids derived from broccoli “TI-111,” we concluded that a better chromosome doubling effect was obtained when the haploid plants were treated with 0.05% colchicine for 6–12 h; the DH frequency ranged from 16.7 to 37.5%, the total chromosome doubling ranged from 54.5 to 75.0%, and the survival rate ranged from 57.1 to 80.0%.

### Evaluation of chromosome doubling in cabbage and broccoli induced by colchicine

According to the experimental results mentioned above, a 0.2% colchicine treatment for 9–12 h was used for cabbage, and a 0.05% colchicine treatment for 9–12 h was used for broccoli. The plant survival rate of the cabbage population “08F9” was low, at 61.9%, but the survival rates for the 11 other populations ranged from 77.3 to 87.0% (Table 4). Among the surviving plants, with the exception of the cabbage population “08F9” and the broccoli population “09B411,” which had low total chromosome doubling rates of only 24.6 and 31.7%, respectively, the five other cabbage populations had total chromosome doubling rates ranging from 42.3 to 57.7%, and those for the five other broccoli populations ranged from 43.8 to 60.0%. It is worth mentioning that a higher frequency of chromosome-doubled genotypes was obtained for all of the haploid genotypes of each population compared with the total chromosome doubling efficiency of the surviving plants. For the cabbage population “08F9” and the broccoli population “09B411,” the frequencies of the chromosome-doubled genotypes were 40.9 and 39.3%, respectively. For the five other cabbage and five other broccoli populations, the frequencies of the chromosome-doubled genotypes ranged from 49.2 to 69.6% and 64.3 to 72.7%, respectively.

**Table 4:** **Application of chromosome doubling in different cabbage and broccoli genotypes induced by colchicine**.

**Materials**		**Number of haploid genotypes**	**Number of plants**	**Number of surviving plants**	**Chromosomal ploidy level**			**Frequency of chromosome doubling (%)**		**Plant survival rate (%)**	**Total chromosome chromosome doubling**	
					**Haploid**	**Diploid**	**Mixed-ploidy**	**Diploid frequency**	**Total chromosome doubling**		**Number of genotypes**	**Frequency of genotypes (%)**
Cabbage	08F8	72	360	288	146	124	18	43.1	49.3	80.0	39	54.2
	08F9	44	210	130	98	28	4	21.5	24.6	61.9	18	40.9
	08F18	44	189	156	66	84	6	53.8	57.7	82.5	30	68.2
	08F24	129	821	660	330	318	12	48.2	50.0	80.4	76	58.9
	08F25	118	682	532	307	211	14	39.7	42.3	78.0	58	49.2
	Zhonggan No. 11	23	92	80	44	40	6	50.0	57.5	87.0	16	69.6
Broccoli	09B411	56	286	243	166	55	22	22.6	31.7	85.0	22	39.3
	10B635	22	122	101	55	32	14	31.7	45.5	82.8	16	72.7
	10B644	17	88	68	36	24	8	35.3	47.1	77.3	11	64.7
	10B908	20	108	89	50	32	7	36.0	43.8	82.4	13	65.0
	10B1092	28	146	114	54	48	12	42.1	52.6	78.1	18	64.3
	12B520	3	24	20	8	11	1	55.0	60.0	83.3	2	66.7

### Effect of tissue culture duration on the chromosome doubling of the population

An experiment was performed to determine whether the tissue culture duration could change the chromosomal ploidy of the microspore-derived regenerants.

In this experiment, 9 populations of cabbage and 3 populations of broccoli were tested, and the ploidy variation of the plantlets was observed during the tissue culture. Notably, the chromosome doubling rate of the population gradually increased with an increase in the tissue culture time (Table 5). After 1 or more years of tissue culture, the number of chromosomes of the haploids doubled, and most of the haploids turned into DHs or mixed-ploidy plants (data not shown).

**Table 5:** **Spontaneous doubling of populations during different tissue culture years**.

**Materials**		**Number of populations**	**Year**			
			**2010**	**2011**	**2012**	**2013**
Cabbage	08F8	106	50.9	66.2	74.3	82.4
	08F9	116	18.1	24.0	38.5	47.8
	08F15	3	0.0	50.0	50.0	100.0
	08F16	2	0.0	25.0	50.0	100.0
	08F17	3	33.3	33.3	67.7	67.7
	08F18	99	68.7	78.6	82.4	96.0
	08F24	380	55.0	76.6	84.4	92.2
	08F25	266	53.8	75.0	82.4	90.0
	Zhonggan 11	145	65.5	77.4	85.2	100.0
Broccoli	09B411	32	–	81.3	93.6	100.0
	10B908	36	–	–	55.6	77.8
	10B1092	36	–	–	61.1	88.9

## Discussion

The chromosome doubling of microspore-derived haploids is an important factor in the practical application of microspore culture technology because breeding programs require a large number of genetically stable, homozygous DH plants with a high level of fertility. Theoretically, a microspore only carries half of the chromosomes of the somatic donor plant, and a plantlet derived from a microspore should be haploid. However, in practice, microspore-derived populations, in addition to haploids, also contain a certain proportion of diploids and even a small number of polyploids. Furthermore, in our experiments, in addition to haploids, diploids and polyploids, a low frequency of mixed-ploidy plantlets (a plant having both haploid and diploid branches simultaneously) was also obtained in cabbage and broccoli. The spontaneous doubling rate varies among crop species and genotypes. In *Brassica napus*, the spontaneous chromosome doubling rates of the microspores range from 10 to 26% (Chen et al., 1994), whereas higher spontaneous doubling rates (50–70%) were observed in Chinese cabbage (Zhang and Takahata, 2001). In broccoli, the diploid rate ranges from 43 to 88%, and in other coles, this rate ranges from 7 to 91% (da Silva Dias, 2003). In our research, the spontaneous diploid rate in cabbage ranged from 0 to 76.9%, and the total spontaneous doubling ranged from 0 to 84.6%. However, in broccoli, the spontaneous diploid rate ranged from 50.6 to 100%, and the total spontaneous doubling ranged from 52.2 to 100% (Table 2). Obviously, there was a larger range in the spontaneous chromosome doubling in the microspore-derived regenerated cabbage populations compared with those of broccoli. This is in good agreement with the results presented by da Silva Dias (2003).

A high proportion of spontaneous DH plants is particularly beneficial in breeding because there is no need to use colchicine to double the haploid chromosomes. Spontaneous doubling saves time and labor. To date, the mechanism of spontaneous chromosome doubling remains unclear, while in the mitotic divisions of microspores, the induction of androgenesis and chromosome doubling both appear to involve changes in microfilaments and microtubules. Microfilaments and microtubules may be responsible for the nuclear migration around the uni-nucleate microspore wall. If a pretreatment system for inducing embryogenesis disrupts the microtubules, this type of treatment might lead to chromosome doubling (Kasha, 2005). Colchicine and a number of other anti-microtubule agents have been used to improve chromosome doubling in microspore cultures of *Brassica* crops. Zhao et al. (1996) and Zhou et al. (2002) found that treatment with colchicine instead of heat shock improved the diploid frequency of microspore-derived *Brassica napus* plants. Li and Devaux (2003) found that cold and mannitol pretreatments of barley microspore cultures resulted in high rates of DHs, regardless of the explant (anther or spike) used. Kasha et al. (2001) concluded that the nuclear fusion of microspores was the main mechanism of spontaneous chromosome doubling in barley isolated microspore cultures following mannitol and cold pretreatments. In our previous study, the combination of cold pretreatment and heat shock resulted in a population with more spontaneous DHs compared with heat shock alone (Yuan et al., 2011).

Furthermore, the microspore culture stage could also influence spontaneous chromosome doubling. For example, culturing of early uni-nucleate stages produced predominantly haploid progeny, whereas culturing of bi-nucleate stages produced more DHs and polyploids (Kasha, 2005). Soriano et al. (2007) found that the spontaneous doubling rates (54–66%) of wheat microspores during the late uni-nucleate to early bi-nucleate stages were higher than those (33%) in the mid to late uni-nucleate stages. This may be related to the late uni-nucleate to early bi-nucleate stages being the best microspore stages for the highest embryo induction (Pechan and Keller, 1988).

Although, several factors can affect spontaneous chromosome doubling in microspores, the same microspore culture stage and type of pretreatment were used for the genotypes of the cabbage and broccoli microspore cultures in our study. Our results indicate that the spontaneous chromosome doubling rate varies among *B. oleracea* crops and genotypes.

da Silva Dias (2003) suggested that is not necessary to induce doubling in genotypes with over 60% spontaneous doubling. In our research, the spontaneous doubling rate of 10 of the 14 microspore-derived populations (71.4%) of cabbage was less than 60% (Table 2). It is clear that the frequency of spontaneous doubling can be very low in cabbage. The spontaneous doubling rate exceeded 60% in most of the broccoli populations in the experiment (Table 2). However, a successful chromosome-doubling process is necessary for genotypes with low spontaneous doubling. Therefore, the development of efficient chromosome-doubling protocols is essential for the useful application of DH plants in cabbage and broccoli breeding programs.

Chromosome doubling can be induced in the early stages of gametic embryogenesis or during the developmental stage of haploid plantlets (Ferrie, 2003). Rudolf et al. (1999) found that *in vitro* treatment with anti-microtubule agents can enhance chromosome doubling, but this method can also reduce embryogenesis (Ferrie, 2003) and regenerant frequency (Hansen and Andersen, 1998a) in microspore cultures. Furthermore, this approach can increase the contamination of microspore cultures. In our study, haploid plantlet roots were immersed in a colchicine solution. This method is simple, convenient and targeted. This procedure produced double chromosomes in over 50% of the haploid genotypes for most of the populations (75%) derived from cabbage and broccoli (Table 4).

Many factors affect the chromosome-doubling process, such as the colchicine concentration, treatment duration, the addition of other synthetic compounds, and the developmental stage of the plants.

A higher colchicine concentration can increase the doubling rate, but a higher concentration also results in a low survival rate and increased cost. Similarly, the duration of colchicine exposure can have a considerable effect on the induction of chromosome doubling and the survival rate. The optimal colchicine treatment should result in a high plant survival rate and a high rate of chromosome doubling. In this study, 0.2% colchicine for 9–12 h or 0.4% colchicine for 3–9 h for cabbage and 0.05% colchicine for 6–12 h for broccoli produced optimal chromosome haploid doubling effects (Table 3).

In our study, the immersion of haploid plantlet roots in a colchicine solution was used to induce chromosome doubling; therefore, the haploid plants used for this purpose must have good root systems. The development of the root systems can affect the colchicine solution absorption efficiency, chromosome doubling and survival rates. Prior to the colchicine treatment, we selected haploid plants with good root systems and then trimmed the roots to better absorb the colchicine solution. Furthermore, 2% DMSO was added to the colchicine solution to increase the root absorption rate.

It is well known that two approaches can be used for chromosome doubling, i.e., spontaneous doubling and artificial chromosome doubling induced by colchicine or other anti-microtubule agents. In our study, which spanned a period of 4 years, we noted that after 1 or more years of tissue culture, the chromosome content of the haploids was doubled, and most of the haploid plants became DHs or mixed-ploidy plants. This phenomenon indicates that the chromosome number of haploids derived from cabbage and broccoli microspores is not stable and can easily be induced to change as a result of external conditions. This is the first report suggesting that tissue culture duration can change the chromosomal ploidy of the microspore-derived regenerants.

## Author contributions

SY performed the experiments, analyzed the data, wrote and revised the manuscript; YS performed the experiments, analyzed the data and revised the manuscript; YL designed the research and critically edited the manuscript; and ZL, ZF, LY, MZ, YZ, HL, and PS planted and managed the plants. All authors approved the final manuscript.

### Conflict of interest statement

The authors declare that the research was conducted in the absence of any commercial or financial relationships that could be construed as a potential conflict of interest.
